# Learning curve of Persona Partial Knee (PPK) arthroplasty: a clinical trial

**DOI:** 10.1186/s12891-024-07215-5

**Published:** 2024-02-10

**Authors:** Riccardo D’Ambrosi, Danko Dan Milinkovic, Filippo Migliorini, Ilaria Mariani, Nicola Ursino, Timothy Hewett

**Affiliations:** 1 IRCCS Ospedale Galeazzi Sant’Ambrogio, Milan, Italy; 2 Dipartimento Di Scienze Biomediche Per La Salute, Università Degli Studi Di Milano, Milan, Italy; 3 Department of Orthopedic and Trauma Surgery, Arcus Sportclinic, Pforzheim, Germany; 4 Department of Orthopaedic, Trauma, and Reconstructive Surgery, RWTH University Medical Centre, Pauwelsstraße 30, 52074 Aachen, Germany; 5 Department of Orthopaedic and Trauma Surgery, Academic Hospital of Bolzano (SABES-ASDAA), Teaching Hospital of the Paracelsus Medical University, 39100 Bolzano, Italy; 6 Institute for Maternal and Child Health IRCCS Burlo Garofolo, Trieste, Italy; 7 Hewett Global Consulting, Rochester, Minneapolis USA

**Keywords:** Unicompartmental, Monocompartmental, Replacement

## Abstract

**Background:**

Unicompartmental knee arthroplasty (UKA) procedures are considered to be more technically demanding than conventional total knee arthroplasty (TKA), requiring a longer learning curve and more expert surgical skills. Despite some clear advantages of UKA over TKA (such as lesser blood loss, greater bone stock, greater knee performances, etc.), UKA evidenced a greater rate of revision.

**Object:**

This study investigated the learning curve of Persona Partial Knee (PPK) arthroplasty for primary medial UKA performed by a single, non-designer surgeon. PPK is a fixed-bearing, compartment-specific implant. The primary outcome of interest for this study was to evaluate the learning curve of the surgical duration. The secondary outcome of interest was to evaluate the learning curve of radiological implant positioning.

**Methods:**

Patients who underwent primary medial UKA using PPK (Zimmer-Biomet, Warsaw IN, USA) were prospectively enrolled for the study. All surgeries were performed by a single, non-designer surgeon experienced in knee and hip arthroplasty. The primary outcome of interest was to evaluate the surgical duration. The secondary outcome of interest was to evaluate the implant positioning. The learning curve was estimated using an appropriate nonlinear polynomial regression model with a lower Akaike Information Criterion (AIC).

**Results:**

One hundred twenty five patients were enrolled in the study. 59% of them (74 of 125 patients) were women. The patients’ mean age at the time of surgery was 70.1 ± 9.5 years and their mean body mass index (BMI) was 27.8 ± 4.2 kg/m^2^. Curve stabilisation of the surgical time was at the 94th patient, of the tibial angle at the 47th patient, of the tibial slope at the 54th patient, of the anterior protrusion at the 29th patient, and of the posterior protrusion at the 51st patient.

**Conclusions:**

The learning curve for component positioning was achieved in approximately 50 cases. The curve of the surgical time achieved a plateau at 94 Persona Partial Knee. Additionally, the factors directly correlated with earlier stabilization of the learning curve in terms of component positioning were: male gender, younger age, right side, and larger components.

**Supplementary Information:**

The online version contains supplementary material available at 10.1186/s12891-024-07215-5.

## Introduction

The upgrade to newly introduced techniques or technology in knee arthroplasty is often challenging, even for experienced surgeons [1]. When considering progression to a new system, surgeons have to undergo a learning curve [2]. The surgical learning curve relates to the improvement of surgical skills and reproducibility of results over time and/or with increasing experience related to a specific surgical technique or technology [3–5]. The surgical learning curve is highly variable between techniques and is strongly related to several individual and institutional factors, including surgeon expertise, surgical team, caseload volume, patient turnover, and type of institution [2]. The duration, magnitude, and variability of the learning curve also have relevance in the surgical outcome [3–5]. For example, shorter surgical durations are associated with a lower risk of infections, shorter anaesthesia, lesser stress for the patient’s body, and cost-effectiveness [2, 6].

During the past few decades, there has been a significant development in the field of knee arthroplasty, and several types of implants, instruments, and surgical techniques have been introduced [7]. Knee arthroplasty provides satisfactory results with a low rate of complications, which has made it one of the most performed orthopaedic surgical procedures worldwide [8, 9]. Although both total knee arthroplasty (TKA) and monocompartmental knee arthroplasty (UKA) are based on similar principles, UKA procedures generally require a longer learning curve and stronger surgical skills, as they are more technically challenging than conventional TKA [10–12]. Further, despite having some clear advantages over TKA (such as lesser blood loss, greater bone stock, greater knee performances, etc.), UKA evidenced a greater rate of revision [13–15].

UKA is typically less invasive than TKA, sparing damage to soft tissues and preserving more bone stock [16, 17]. There is a growing consensus among surgeons that UKA is associated with better clinical and functional performances than TKA [16, 17]. Moreover, UKA is associated with lower blood loss, shorter surgical duration, and a shorter period of hospitalization. UKA also allows a quicker return to high-level sports when compared to TKA [16, 17]. However, one major downside of UKA is that it is associated with reduced implant survivorship when compared to TKA.

Nevertheless, most of the early failures of UKA are related to technical pitfalls. UKA has special instrumentation and a longer learning curve when compared to TKA [18, 19]. Therefore, many surgeons remain reluctant to adopt UKA as their new arthroplasty system.

Few studies investigated the learning curve of single-compartment arthroplasty. However, they have used different evaluation criteria and have therefore reported different learning curves [3, 11, 20]. Our study investigated the radiological positioning of each component, in connection to surgical times and complications. This study investigated the learning curve of Persona Partial Knee (PPK) arthroplasty for primary medial UKA performed by a single, non-designer surgeon. PPK is a fixed-bearing, compartment-specific implant. The primary outcome of interest for this study was to evaluate the learning curve of the surgical duration. The secondary outcome of interest was to evaluate the learning curve of radiological implant positioning. It was hypothesised that less than a hundred procedures should be required to overcome the learning curve.

## Materials and Methods

### Study protocol

In this study, all the procedures involving human participants were performed in compliance with the 1964 Helsinki Declaration and its later amendments. The present study was approved by the Ethics Committee of the University of Milan, Italy (CE 236/2017). The study was conducted by following the Strengthening the Reporting of Observational Studies in Epidemiology: the STROBE checklist [21]. Informed consent was obtained from all the participants. Patients who underwent primary UKA using PPK (Zimmer-Biomet, Warsaw IN, USA) were prospectively enrolled in the study between January 2018 and June 2021. All procedures were performed by a single, non-designer surgeon experienced in knee and hip arthroplasty, blinded to the purpose of the study.

### Eligibility criteria

The inclusion criteria were: (1) end-stage isolated knee osteoarthrosis of the medial compartment, (2) avascular necrosis of the medial femoral condyle, (3) varus or valgus deformity < 3°, (4) range of motion (ROM) > 100°, (5) less than 10° of extension contracture, (6) ligaments integrity, (7) minimum follow-up of 12 months; (8) Body Mass Index (BMI) < 30 kg/m^2^. Magnetic resonance imaging (MRI) was conducted preoperatively to confirm the integrity of cruciate and collateral ligaments in all patients (this procedure is routinely performed before the UKA implant). The exclusion criteria were: (1) previous knee surgery (except arthroscopy for meniscectomy), (2) implant failure during follow-up.

### Surgical technique

All patients were operated on under spinal anesthesia in a supine position, by a single, non-designer surgeon experienced in knee and hip arthroplasty*.* No tourniquet was used. A standard mini-medial parapatellar approach was used for all patients. A routine inspection of the patellofemoral and lateral compartments was conducted. The integrity of the cruciate ligament was evaluated. The medial and intercondylar osteophytes were removed. An anterior tibial pre-cut was performed to allow articular exposure. The PPK system (Zimmer, Warsaw, Indiana, USA) was implanted using the corresponding instrumentation, extramedullary tibial guide, and the femoral and tibial cutting guides by following manufacturer instructions [22]. All the components were cemented using Refobacin® Bone Cement R (Zimmer-Biomet, Warsaw, Indiana, USA). One closed suction subcutaneous drain was used and removed on the first postoperative day. The postoperative rehabilitation protocol was identical for all patients and followed published guidelines [23].

### Outcomes of interest

The primary outcome of interest was to evaluate the surgical duration. Surgical duration is the time from skin incision to wound suture. The secondary outcome of interest was to evaluate the implant positioning. Implant positioning was assessed independently by two joint replacement specialists who were not involved in the clinical management of the patients. The radiologic measurements were performed twice, at an interval of four to six weeks.

**Fig. 1:**
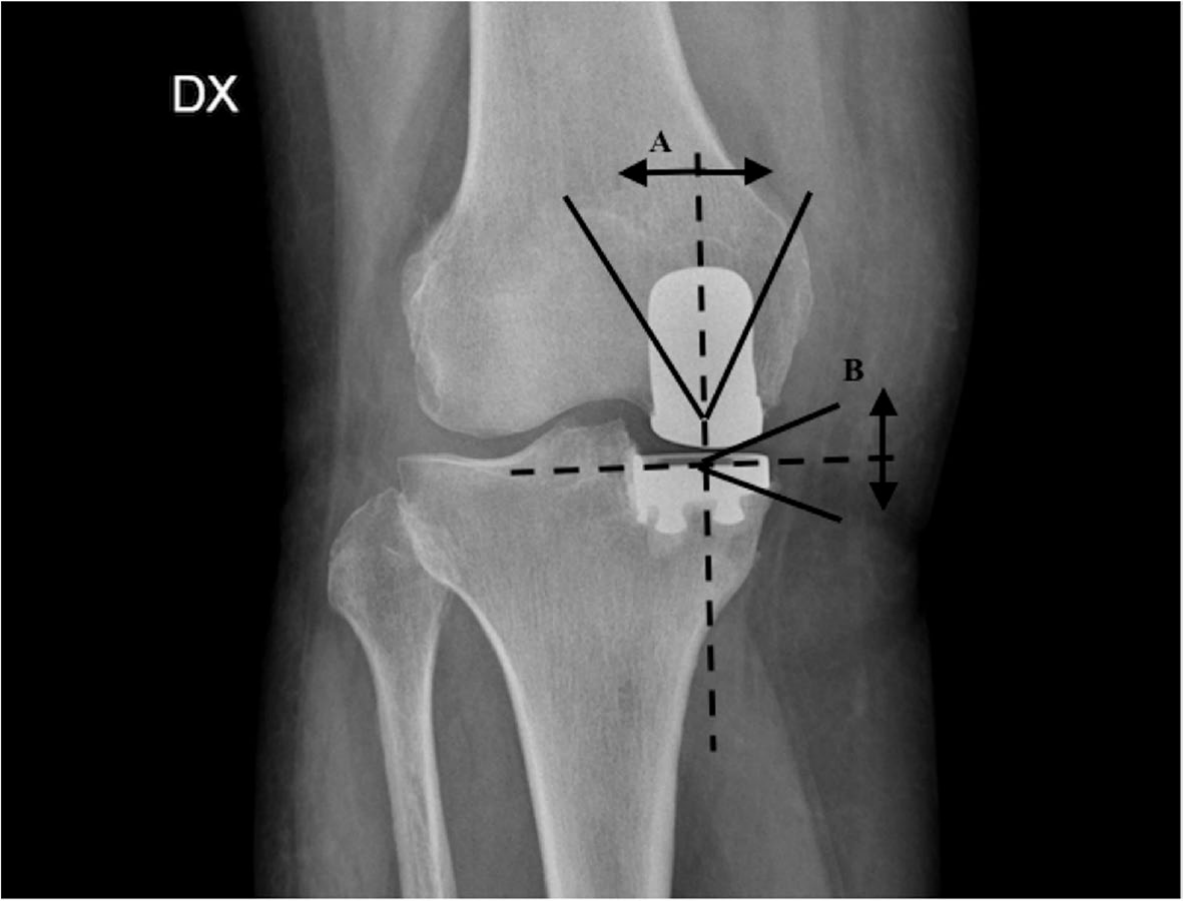
Antero-posterior radiograph, right knee; Radiological parameters measured: femoral varus/valgus angle (**A**); tibial varus/valgus angle (**B**)

**Fig. 2:**
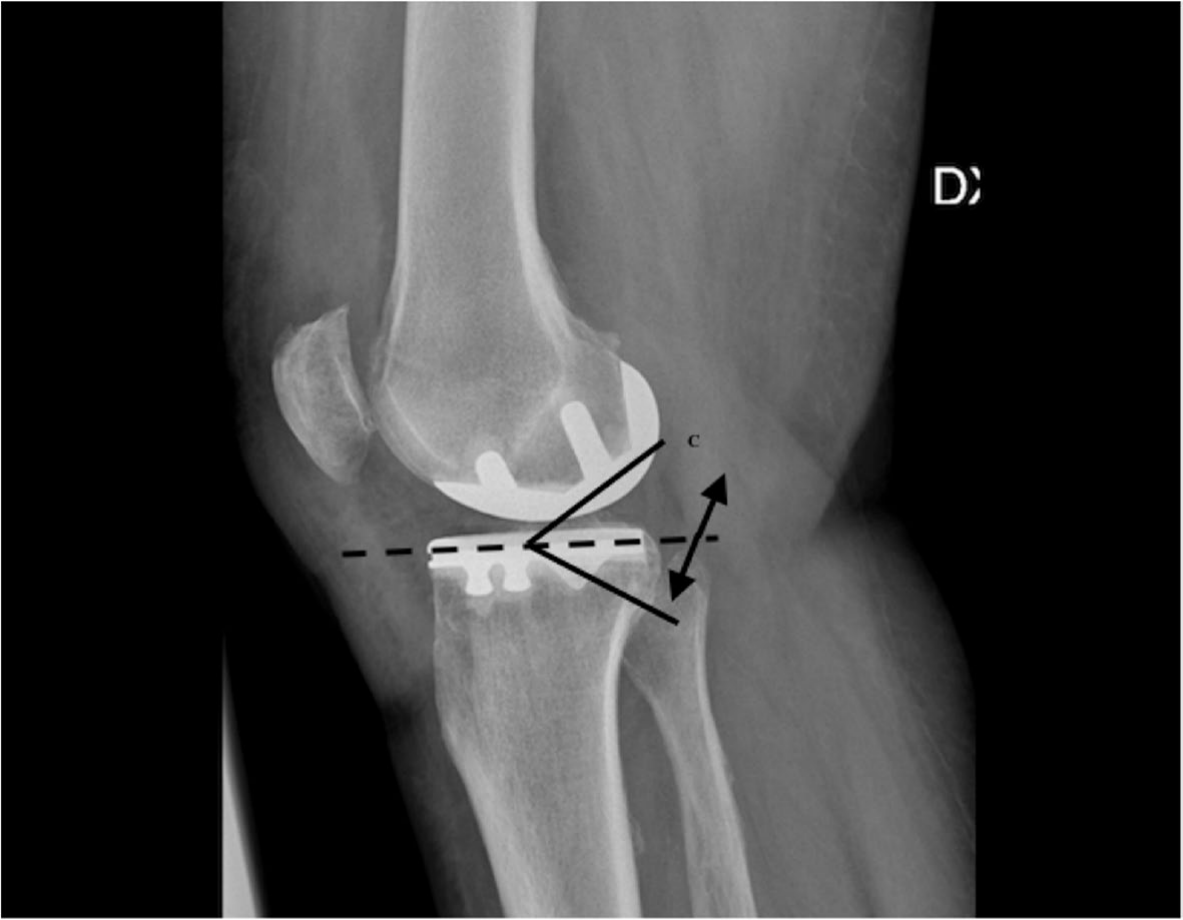
Lateral radiograph, right knee; Radiological parameters measured: anteroposterior tibial slope (**C**)

The implant positioning was evaluated using the following parameters at radiographs [24–26] (Figs. 1 and 2):

- Femoral component varus/valgus: Angle between the femoral component and the femoral axis in the coronal plane. An angle of 7° was considered neutral with a tolerance range of ± 10°
- Tibial component varus/valgus: Angle between the tibial axis and a line drawn along the tibial tray in the coronal plane. The range of tolerance was ± 5°
- Anteroposterior slope: Angle between a line drawn along the tibial tray and perpendicular to the tibial axis in the lateral view. A slope of 7° was considered optimal with a tolerance range of ± 5° (2° to 12°)
- Cortex perforation due to tibial peg position
- Tibial spine integrity
- Medial tibial component protrusion
- Anterior tibial component protrusion
- Posterior tibial component protrusion

Radiological data was extracted from Digital Imaging and Communications in Medicine (DICOM) to Picture Archiving and Communications System (PACS) and collected in OsiriX® imaging software (version 4.1.2 32-bit) [27].

### Statistical analysis

The sociodemographic, clinical, and surgical characteristics of the samples were described using absolute frequencies and percentages for binary data and mean and standard deviations for continuous data. To evaluate the association between the cumulative volume of cases and variables, Spearman or Pearson correlation coefficients were estimated and tested. To assess the learning curve of implant positioning and surgical duration, a moving average with subgroups of 20 consecutive patients was calculated. The learning curve was estimated using the appropriate nonlinear polynomial regression model with a lower Akaike information criterion (AIC) and a statistically significant reduction in the residual sum of squares compared to models with a different degree. Lower AIC means that a model should have improved prediction. Frequently adding more variables decreases predictive accuracy, and the model with a higher R2 will express higher (worse) AIC. To evaluate the presence of a significant trend, a Mann–Kendall test was performed for each variable. Further, to identify the point at which the learning curve stabilized, piecewise linear regression with iterative breakpoint estimation was performed using the local maximum or minimum of the nonlinear polynomial regression as the initial breakpoint. To assess whether the learning curves were influenced by sociodemographic and clinical characteristics (such as sex, age, BMI, surgical side, tibial size, femoral size, and dichotomizing continuous variables at their average), a t-test was conducted by adding the aforementioned variables to the previously estimated models. Then, a subgroup analysis was conducted to study this learning curve within patient subgroups with similar sociodemographic and clinical characteristics.

## Results

### Patient recruitment

**Fig. 3:**
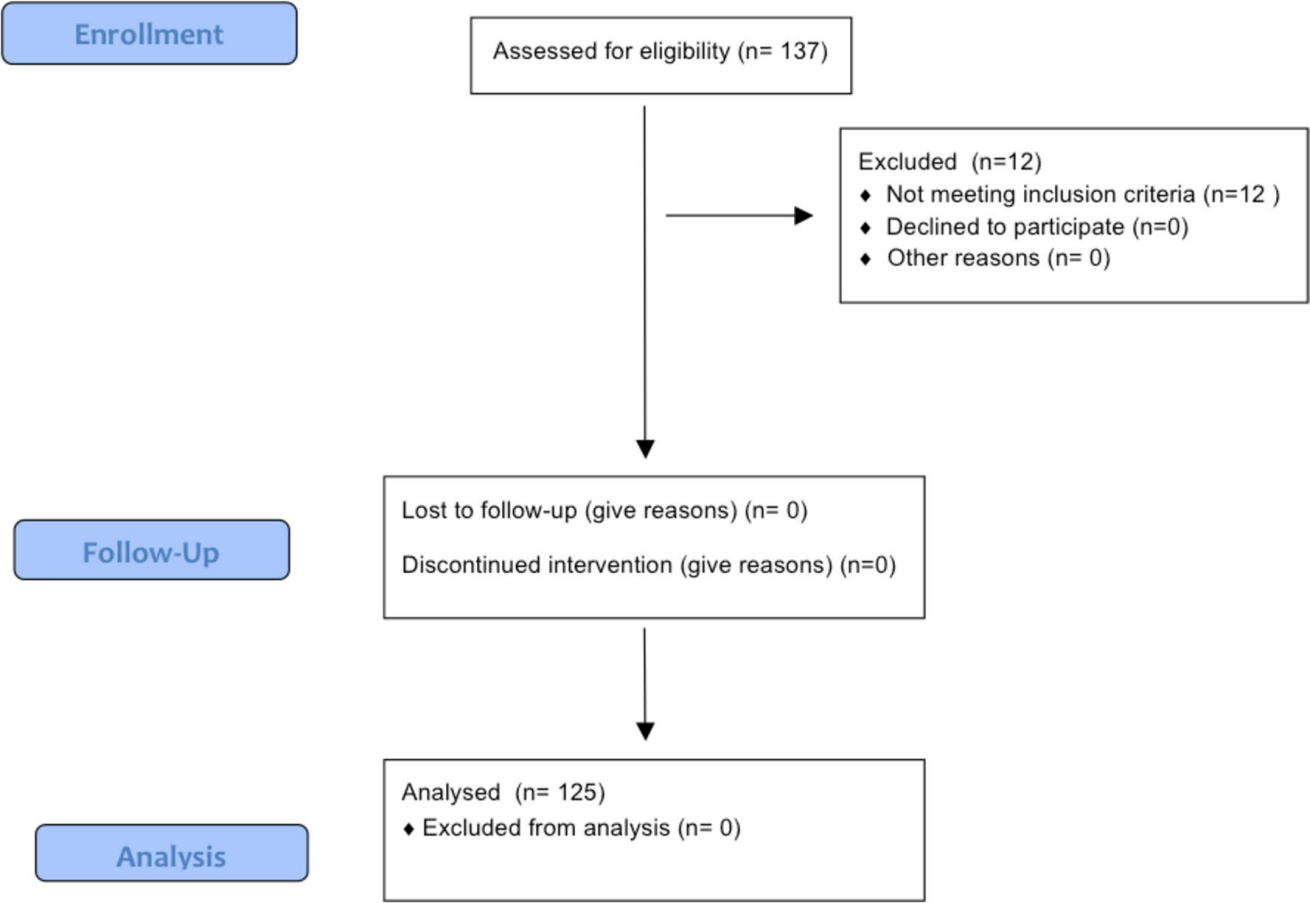
STROBE diagram of patient recruitment

A total of 137 patients were initially screened. Of them, 12 were not eligible, owing to previous high tibial osteotomy (*N* = 7) and previous femoral/tibial fracture (*N* = 5). After removing these patients, 125 patients were enrolled in the study. No patients were lost during follow-up (Fig. 3).

### Demographic and surgical data

Notably, 59% of the patients (74 of 125 patients) were women. The patients’ mean age at the time of surgery was 70.1 ± 9.5 years and their mean BMI was 27.8 ± 4.2 kg/m^2^. Demographic data is presented in greater detail in Table 1.

**Table 1:** Demographics of patients included in the study

Variable	Value
Year	
2018	2 (1.6%)
2019	21 (16.8%)
2020	43 (34.4%)
2021	59 (47.2%)
Sex	
F	74 (59.2%)
M	51 (40.8%)
Age *[mean* ± *SD]*	70.10 ± 9.48
BMI *[mean* ± *SD]*	27.76 ± 4.23
Side	
Right	61 (48.8%)
Left	64 (51.2%)
Tibial size	
C	2 (1.6%)
D	27 (21.6%)
E	38 (30.4%)
F	19 (15.2%)
G	19 (15.2%)
H	14 (11.2%)
J	6 (4.8%)
Femoral size	
1	3 (2.4%)
2	13 (10.4%)
3	29 (23.2%)
4	32 (25.6%)
5	26 (20.8%)
6	14 (11.2%)
7	8 (6.4%)
Surgical time (minutes)	47.27 ± 9.71
Tibial angle (°)	-1.38 ± 1.95
Femoral angle (°)	0.23 ± 2.50
Tibial slope (°)	4.48 ± 2.11
Anterior protrusion (mm)	0.05 ± 0.44
Posterior protrusion (mm)	-0.15 ± 0.74
Medial protrusion (mm)	-0.02 ± 0.76
Bearing (thickness)	8.38 ± 0.68
Intact tibial spine	
No	1 (0.8%)
Yes	124 (99.2%)
Cortex perforation	
No	122 (97.6%)
Yes	3 (2.4%)

### BMI Body mass index

## Overall learning curve

Stabilization was achieved for the surgical time at the 94th patient, for the tibial angle at the 47th patient, for the tibial slope at the 54th patient, for the anterior protrusion at the 29th patient, and for the posterior protrusion at the 51st patient. These results are shown in greater detail in Table 2.

**Table 2:** Overall Learning Curve

	***Moving average* ± *SD***					**Mann–Kendall trend test* Value (p-value)***	**Learning curve**
	**1st patient group**	**25th patient group**	**50th patient group**	**75th patient group**	**106th patient group**		
Surgical time	47.8 ± 9.42	49.3 ± 9.85	50.4 ± 9.58	48.3 ± 9.17	42.6 ± 10.8	-0.15 (p = 0.01)	The learning curve stabilized after the 94th patient group
Tibial angle	-2.85 ± 2.03	-2.75 ± 2.71	-0.65 ± 1.27	-0.85 ± 1.27	-0.6 ± 0.94	0.30 (p < 0.001)	The learning curve stabilized after the 47th patient group
Femoral angle	0.60 ± 3.47	-0.60 ± 3.15	0.55 ± 2.52	0.05 ± 1.32	-0.45 ± 1.64	-0.05 (p = 0.4)	No learning curve
Tibial slope	3.90 ± 2.51	2.50 ± 2.80	4.75 ± 1.25	5.3 ± 1.34	5.50 ± 0.83	0.35 (p < 0.001)	The learning curve stabilized after the 54th patient group
Anterior protrusion	0.35 ± 0.59	-0.05 ± 0.61	0 ± 0.32	-0.1 ± 0.45	0 ± 0.0	-0.21 (p = 0.003)	The learning curve stabilized after the 29th patient group
Posterior protrusion	-0.70 ± 1.13	-0.35 ± 0.99	0.10 ± 0.45	-0.05 ± 0.51	-0.10 ± 0.31	0.23 (p < 0.001)	The learning curve stabilized after the 51th patient group
Medial protrusion	0.0 ± 0.86	-0.15 ± 1.04	0.0 ± 0.32	0.10 ± 0.64	-0.25 ± 0.72	-0.06 (p = 0.05)	No learning curve
Bearing (thickness)	8.55 ± 0.76	8.40 ± 0.60	8.55 ± 0.61	8.00 ± 0.00	8.5 ± 1.05	-0.09 (p = 0.2)	No learning curve

### Learning curve by gender

In male patients, we noted an earlier stabilization of the tibial angle and tibial slope when compared to the female patients (18th versus 25th and 21st versus 33rd). These results are shown in greater detail in Appendix Table S1.

### Learning curve by age

In younger patients (< 70 years), we found an earlier stabilization of the curves for tibial angle and tibial slope when compared to older patients (≥ 70 years) (18th versus 24th and 18th versus 36th). These results are shown in greater detail in Appendix Table S2.

### Learning Curve by BMI

Patients with BMI < 27 showed an earlier stabilization in the tibial angle (18th versus 24th) but a similar stabilization in the tibial slope (26th versus 25th). These results are shown in greater detail in Appendix Table S3.

### Learning curve by side

Right side knee showed an earlier stabilization with regards to tibial angle (13th versus 27th) and tibial slope (23rd versus 33rd). These results are shown in greater detail in Appendix Table S4.

### Learning curve by tibial size

Small tibial size showed a later stabilization when compared to big tibial size concerning the tibial slope (33rd versus 17th). These results are shown in greater detail in Appendix Table S5.

### Learning curve by femoral size

Small femoral size showed a later stabilization of the curve with regards to tibial angle (22nd versus 20th) and tibial slope (35th versus 20th). Detailed results are reported in Appendix Table S6.

## Discussion

The main findings of our study report that the learning curve for component positioning was achieved in approximately 50 cases. The curve of the surgical time achieved a plateau at 94 PPK.

The surgical learning curve is the improvement of surgical skills and reproducibility of results over time and/or with increasing experience related to a specific surgical technique or technology [4, 5]. The starting point of the curve is influenced by baseline knowledge, and the trend of the curve indicates the accumulated expertise during surgery. The time necessary to attain the expected expert level characterized by reproducibility and consistency of results is indicated by the plateau [2–6]. The learning curve for arthroplasty surgery can be complicated as it involves several variables, including surgical competence, experience, and case volume. Typically, improvements are initially seen quickly until a steady state is reached and a sigmoid-shaped curve is formed. It is complex and challenging to evaluate a specific surgeon’s learning curve for a particular procedure or new technology.

The direct anterior approach (DAA) to total hip arthroplasty is highlighted as a well-studied example of a learning curve in joint replacement surgery. During the past few decades, this technique has become increasingly popular in North America. Research has shown that DAA can improve early functional recovery [28, 29], decrease the duration of hospital stay [30], and raise the rates of early discharge from the hospital. Although there is also literature describing the downsides, many surgeons actively advertise the DAA [30]. Encouraged by this advocacy, several surgeons who are comfortable with alternate techniques to total hip arthroplasty have transitioned to the DAA and encountered troubles with the change early on. Published data reports that the learning curve of transitioning to the DAA ranges from 20 to 50 cases [31, 32], and for departments transitioning together, the number is cited as between 200 and 300 [33]. Although it is useful to know at what number of cases the steady state can be attained, surgeons must examine the results of the initial patients and the higher risks for adverse events in that cohort to determine whether the additional risk to those patients warrants the change. However, even though expertise and efficacy gradually increase over time, no clear criteria or consensus exists on how to determine and report learning curves in TKA and UKA.

The purpose of this study was to establish the learning curve for PPK and explore the impact of several demographic and technical factors on the curve itself. Our analysis showed that the stabilization of the learning curve starts approximately after the first 50 procedures, and the surgical time stabilizes around the 100th case. In terms of coherent component positioning, according to previously established radiographic trash-holds and criteria proposed by the Zimmer Biomet Instructional manual for PPK arthroplasty system [22], demographic factors such as male gender, younger age, right side, and larger tibial and femoral components, are associated with a faster stabilization of the learning curve.

In medial PKA, we aim to achieve a slight mechanical varus (3–5°) alignment to increase the success and durability of the implant. Patients with well-functioning UKAs at 10 years exhibited mild varus mechanical alignment of approximately 4°, whereas patients revised for progression of osteoarthritis averaged more valgus and those revised for loosening or subsidence averaged more varus [34–36].

Given the paucity of available data on learning curves related to UKAs and TKAs, and the heterogeneous methodology of relevant studies, comparison of different learning curves is arduous. For example, Zhang et al. [37] evaluated 50 consecutive cases of minimally invasive Oxford Phase III UKA performed by a single surgeon at a mean of 51 months of follow-up to determine whether there was an association between outcomes and the cumulative number of cases performed. Converse to our study, the recorded data was analysed using the cumulative summation test for learning curve (LC-CUSUM), which showed that failure rates diminished rapidly after 16 cases and reached an acceptable rate after 29 cases [37]. The slope of the curve, the duration of surgery, blood loss, and length of incision improved gradually with the cumulative number of procedures alike [37]. Minimally invasive Oxford Phase III UKA is a demanding surgical procedure; however, satisfactory outcomes can be achieved after 25 consecutive cases.

Kayani et al. determined a surgical team’s learning curve for introducing a robotic-arm-assisted UKA into routine surgical practice [11]. The single-surgeon cohort study included 60 consecutive conventional jig-based UKAs compared with 60 consecutive robotic-arm assisted UKAs for medial compartment knee osteoarthritis. Robotic-arm-assisted UKA was associated with a learning curve of six cases for operating time (p < 0.001) and surgical team confidence levels (p < 0.001). The cumulative robotic experience did not affect the accuracy of implant positioning, posterior condylar offset ratio, posterior tibial slope (p = 0.7), native joint line preservation, and postoperative limb alignment. The robotic-arm-assisted UKA improved the accuracy of femoral (p < 0.001) and tibial (p < 0.001) implant positioning with no additional risk of postoperative complications compared to conventional jig-based UKA [11].

Hamilton et al. examined 445 consecutive minimally invasive UKAs to determine whether revision and reoperation rates decreased as the number of cases performed increased, indicating the presence of a learning curve for this procedure. For the first half of UKA cases performed versus the second half, revision rates fell from 5.0% to 2.5%, and reoperation rates fell from 8.1% to 5.4%, demonstrating that despite modifications made to improve surgical techniques across time, a substantial complication rate persists in this procedure [20].

In our clinical experience, PKA is implanted in 10–20% of cases, a slightly higher percentage than in registries [7]. This number is due to the high surgical volume at our institution along with long experience which is a key factor for more satisfactory surgical results [25, 38–40].

In the present study, we found a deeper learning curve, which can be attributed to several factors. Apart from the surgical time and failure rate, we also analysed the surgical time, failure rate, radiographic irregularities (cortex perforation and tibial spine integrity), and correct positioning of the components. We also split the patients by sex, age, BMI, surgical side, tibial size, and femoral size. So, to our knowledge, this is the most comprehensive study that evaluates multiple variables for defining learning curves for single-compartment prostheses.

Learning curves involved in the use of new technologies and devices in joint replacement surgery are a barrier to adoption as they impede surgical workflow for operators and their assistants. This temporary obstruction to workflow must be carefully balanced when new technologies are under consideration to ensure safe care delivery and positive patient clinical outcomes. Establishing learning curves can be highly helpful for potential adopters to understand and forecast how long it may take for them to become skilled in utilizing a new technology and decide whether or not to employ the new technology. Continued research of learning curves associated with new orthopaedic technologies may better guide the decision-making process of device adoption [5].

PPK is a fixed-bearing UKA that was introduced in February 2017. Since then, it has demonstrated good preliminary outcomes. A recent multicentric study investigating PPK in 598 patients reported excellent outcomes in patient-reported outcome measurements (PROMs), patient satisfaction, and imaging, with no cases of revisions during the one-year follow-up [41]. Moreover, this study underlined that the use of PPK is challenging for inexperienced surgeons, so the learning curve may have impacted the outcomes [41].

There are several limitations to our study. The limited follow-up may jeopardize the ability to detect rare complications, in particular, few cases (< 20%) were performed in the first two years of the study (2018 and 2019), lengthening the learning curve. The surgeon who performed the surgeries was well ahead of the learning curve in TKA. Therefore, our results may not apply to young or inexperienced surgeons. The present study was conducted in a highly specialized orthopaedic hospital. In this respect, the results, particularly the surgical time, might be affected by several factors including caseload, financing, the experience of assistants, instrument nurses, and anaesthesiology teams. Potential ethnic differences were not considered when evaluating positioning. Recent studies demonstrated that among ethnicities, African Americans/Blacks, and Asian Americans have increased PTS in comparison with Whites [24, 25]. Nearly 25% of individuals have clinically significant slopes of < 6° or > 12°, with no difference in tibial slope among sex or age groups [42]. Finally, the rotational alignment of the components was not evaluated. The rotational alignment is evaluated using CT imaging which exposes patients to radiation. Excessive external rotation and mismatch between the femoral and tibial components might lead to suboptimal kinematics, reducing contact area and compromised durability after PKA. Finally, the rotational alignment of the components was not evaluated; to do so would have required CT scans of all patients by subjecting them to radiation exposure. Excessive external rotation and mismatch between the femoral and tibial components might lead to suboptimal kinematics, reducing contact area and compromising durability after PKA [43].

## Conclusions

The learning curve for component positioning was achieved in approximately 50 cases. The curve of the surgical time achieved a plateau at 94 Persona Partial Knee. Additionally, the factors directly correlated with earlier stabilization of the learning curve in terms of component positioning were male gender, younger age, right side, and larger components.

### Supplementary Information

**Additional file 1. **

## Data Availability

All data and materials are available on reasonable request to Dr. Riccardo D’Ambrosi (riccardo.dambrosi@hotmail.it).

## Abbreviations

PPK: Persona Partial Knee
UKA: Unicompartmental knee arthroplasty
TKA: Total knee arthroplasty
AIC: Akaike Information Criterion
