# Supplementary material for: Learning curve of Persona Partial Knee (PPK) arthroplasty: a clinical trial
Source: BMC Musculoskelet Disord. 2024 Feb 10;25:128. doi: 10.1186/s12891-024-07215-5 (PMC10858461; doi:10.1186/s12891-024-07215-5)
Supplement: Supplementary file 1 — Additional file 1. [file 12891_2024_7215_MOESM1_ESM.docx]

Appendix

**Table S1.** Learning curve by sex: male (n=51, 31 patient groups) and female (n=74, 55 patient groups).

|  | ***Mean ±SD*** | | | | | **Between group test**  ***Mean difference (p-value)*** | **Subgroup analysis** | |  |
| --- | --- | --- | --- | --- | --- | --- | --- | --- | --- |
|  | **1^st^ patient group** | **15^th^ patient group** | **30^th^ patient group** | **45^th^ patient group** | **last patient group** |  | **Mann–Kendall trend test *Value (p-value)*** | **Learning curve** |  |
| **Surgical time** | | | | | | | | | |
| Male | 52.8 ±10.5 | 51.7 ±9.46 | 47.6 ±11.0 | - | 45.4 ±11.3 | 3.34 (p=0.051) | -0.23 (p=0.016*) | Linear decrease of 0.22 (SE=0.01) for each patient group |  |
| Female | 47.1 ±8.18 | 50.2 ±7.85 | 43.8 ±8.17 | 43.8 ±8.17 | 41.4 ±8.77 |  | -0.11 (p=0.171) | No learning curve |  |
| **Tibial angle** | | | | | | | | | |
| Male | -2.45 ±2.44 | -1.1 ±2.22 | -0.2 ±0.70 | - | -0.04 ±0.88 | 0.05 (p=0.871) | 0.31 (p=0.004*) | The learning curve stabilized after the 18th patient group |  |
| Female | -3.2 ±2.21 | -3.2 ±2.21 | -1.95 ±2.52 | -0.6 ±1.14 | -0.75 ±0.91 |  | 0.28 (p=0.001*) | The learning curve stabilized after the 25th patient group |  |
| **Femoral angle** |  |  |  |  |  |  |  |  |  |
| Male | 0.2 ±3.0 | -0.3 ±1.81 | 0.1 ±1.48 | - | -0.05 ±1.64 | -0.11 (p=0.809) | -0.11 (p=0.269) | No learning curve |  |
| Female | 0.4 ±3.82 | 0.6 ±3.23 | 0.45 ±2.44 | 0.25 ±1.62 | 0.45 ±1.61 |  | -0.01 (p=0.905) | No learning curve |  |
| **Tibial slope** |  |  |  |  |  |  |  |  |  |
| Male | 3.05 ±3.00 | 4.35 ±2.23 | 5.15 ±1.35 | - | 5.3 ±1.26 | -0.23 (p=0.491) | 0.30 (p=0.004*) | The learning curve stabilized after the 21th patient group |  |
| Female | 3.75 ±2.47 | 3.15 ±2.54 | 4.7 ±1.30 | 5.8 ±0.70 | 5.8 ±0.62 |  | 0.38 (p<0.001*) | The learning curve stabilized after the 33th patient group |  |
| **Anterior protrusion** |  |  |  |  |  |  |  |  |  |
| Male | 0.2 ±0.52 | -0.05 ±0.22 | -0.1 ±0.45 | - | -0.1 ±0.45 | 0.002 (p=0.974) | -0.33 (p=0.005*) | The learning curve stabilized after the 18th patient group |  |
| Female | 0.25 ±0.55 | -0.05 ±0.61 | 0.05 ±0.39 | 0.05 ±0.22 | 0.0 ±0 |  | -0.14 (p=0.154) | No learning curve |  |
| **Posterior protrusion** |  |  |  |  |  |  |  |  |  |
| Male | -0.7 ±1.13 | -0.35 ±0.81 | -0.05 ±0.22 | - | -0.05 ±0.22 | -0.32 (p=0.009*) | 0.28 (p=0.013*) | Linear increase of 0.01 mm (SE=0.002) for each patient group |  |
| Female | -0.35 ±0.99 | 0.1 ±0.72 | 0.15 ±0.49 | 0.2 ±0.52 | 0.1 ±0.55 |  | 0.22 (p=0.021*) | The learning curve stabilized after the 24th patient group |  |
| **Medial protrusion** |  |  |  |  |  |  |  |  |  |
| Male | -0.35 ±0.88 | -0.15 ±0.49 | -0.25 ±085 | - | -0.35 ±0.93 | -0.41 (p=0.002*) | 0.04 (p=0.707) | No learning curve |  |
| Female | 0.1 ±0.91 | 0.45 ±0.89 | 0.0 ±0.46 | 0.05 ±0.39 | 0.0 ±0.32 |  | -0.12 (p=0.196) | No learning curve |  |
| **Bearing (thickness)** | Quadratic |  |  |  |  |  |  |  |  |
| Male | 8.3 ±0.57 | 8.35 ±0.59 | 8.5 ±1.05 | - | 8.5 ±1.05 | 0.06 (p=0.620) | 0.06 (p=0.588) | No learning curve |  |
| Female | 8.65 ±0.75 | 8.2 ±0.41 | 8.35 ±0.49 | 8.15 ±0.37 | 8.3 ±0.57 |  | -0.19 (p=0.050) | No learning curve |  |

*=statistical significant value (p<0.05).

**Table S2.** Learning curve by age: young (<70 yrs, n=53, 34 patient groups) and old (≥70 yrs n=72, 53 patient groups).

|  | ***Mean ±SD*** | | | | | **Between group test**  ***Mean difference (p-value)*** | **Subgroup analysis** | |
| --- | --- | --- | --- | --- | --- | --- | --- | --- |
|  | **1^st^ patient group** | **15^th^ patient group** | **30^th^ patient group** | **45^th^ patient group** | **last patient group** |  | **Mann–Kendall trend test *Value (p-value)*** | **Learning curve** |
| **Surgical time** |  |  |  |  |  |  |  |  |
| <70 yrs | 50.8 ±9.08 | 53.2 ±7.67 | 44.1 ±9.52 | - | 42.4 ±10.3 | Ref | -0.21 (p=0.033*) | Significant decrease after the 15^th^ patient group, no curve stabilization |
| ≥70 yrs | 49.0 ±8.41 | 49.0 ±8.41 | 47.8 ±8.75 | 44.7 ±9.41 | 44.0 ±9.77 | -1.21 (p=0.477) | -0.09 (p=0.257) | No learning curve |
| **Tibial angle** |  |  |  |  |  |  |  |  |
| <70 yrs | -3.1 ±2.81 | -1.14 ±2.01 | -0.65 ±0.99 | - | -0.7 ±1.08 | Ref | 0.34 (p=0.001*) | The learning curve stabilized after the 18th patient group |
| ≥70 yrs | -2.45 ±1.82 | -1.55 ±1.27 | -0.35 ±1.1 | -0.6 ±1.10 | -0.55 ±0.76 | 0.062 (p=0.055) | 0.27 (p=0.002*) | The learning curve stabilized after the 24th patient group |
| **Femoral angle** |  |  |  |  |  |  |  |  |
| <70 yrs | 0 ±3.18 | -0.35 ±1.98 | 0.15 ±1.66 | - | -0.1 ±1.86 | Ref | -0.03 (p=0.756) | No learning curve |
| ≥70 yrs | 0.7 ±3.60 | -0.5 ±3.10 | 0.65 ±2.28 | 0.35 ±1.31 | 0.55 ±1.39 | 0.52 (p=0.256) | -0.07 (p=0.39) | No learning curve |
| **Tibial slope** |  |  |  |  |  |  |  |  |
| <70 yrs | 3.35 ±2.8 | 4.2 ±2.42 | 5.4 ±1.35 |  | 5.25 ±1.29 | Ref | 0.30 (p=0.003*) | The learning curve stabilized after the 18th patient group |
| ≥70 yrs | 3.15 ±2.78 | 3.2 ±2.53 | 4.85 ±1.31 | 5.8 ±0.62 | 5.7 ±0.87 | -0.07 (p=0.836) | 0.39 (p<0.001) | The learning curve stabilized after the 36th patient group |
| **Anterior protrusion** |  |  |  |  |  |  |  |  |
| <70 yrs | 0.25 ±0.55 | 0.05 ±0.39 | -0.05 ±0.51 | - | -0.1 ±0.45 | Ref | -0.27 (p=0.019*) | Linear decrease of 0.01 mm (SE=0.002) for each patient group |
| ≥70 yrs | 0.2 ±0.52 | -0.15 ±0.49 | -0.05 ±0.22 | 0 ±0 | 0 ±0 | -0.06 (p=0.392) | -0.16 (p=0.093) | No learning curve |
| **Posterior protrusion** |  |  |  |  |  |  |  |  |
| <70 yrs | -0.65 ±0.81 | -0.15 ±0.75 | 0.05 ±0.22 | - | 0 ±0 | Ref | 0.36 (p=0.001*) | The learning curve stabilized after the 23th patient group |
| ≥70 yrs | 0 ±1.27 | 0.15 ±0.67 | 0 ±0.65 | 0 ±0.76 | 0.05 ±0.61 | 0.11 (p=0.843) | 0.11 (p=0.256) | No learning curve |
| **Medial protrusion** |  |  |  |  |  |  |  |  |
| <70 yrs | -0.1 ±0.97 | -0.05 ±0.61 | -0.05 ±0.76 | - | -0.3 ±0.92 | Ref | -0.042 (p=0.706) | No learning curve |
| ≥70 yrs | -0.1 ±0.91 | 0.2 ±1.01 | 0 ±0.46 | 0 ±0.32 | -0.05 ±0.39 | 0.19 (p=0.148) | -0.08 (p=0.418) | No learning curve |
| **Bearing (thickness)** |  |  |  |  |  |  |  |  |
| <70 yrs | 8.6 ±0.75 | 8.25 ±0.44 | 8.2 ±0.62 | - | 8.2 ±0.92 | 0.04 (p=0.744) | -0.29 (p=0.009*) | Linear decrease of 0.01 (SE=0.001) for each patient group |
| ≥70 yrs | 8.35 ±0.59 | 8.3 ±0.57 | 8.45 ±0.61 | 8.25 ±0.44 | 8.6 ±0.99 | Ref | 0.06 (p=0.521) | No learning curve |

*=statistical significant value (p<0.05). Ref= reference group.

**Table S3.** Learning curve by BMI: BMI < 27 (n=56, 37 patient groups) and BMI ≥ 27 (n=69, 50 patient groups).

|  | ***Mean ±SD*** | | | | | **Between group test**  ***Mean difference (p-value)*** | **Subgroup analysis** | |
| --- | --- | --- | --- | --- | --- | --- | --- | --- |
|  | **1^st^ patient group** | **15^th^ patient group** | **30^th^ patient group** | **45^th^ patient group** | **last patient group** |  | **Mann–Kendall trend test *Value (p-value)*** | **Learning curve** |
| **Surgical time** |  |  |  |  |  |  |  |  |
| BMI < 27 | 50 ±10.3 | 51.2 ±9.02 | 45.4 ±10.5 | - | 43.4 ±10.0 | Ref | -0.18 (p=0.058) | No learning curve |
| BMI ≥ 27 | 48 ±9.22 | 49.4 ±9.10 | 47.9 ±8.60 | 45.2 ±10.8 | 43.3 ±10.6 | -0.43 (p=0.799) | -0.12 (p=0.158) | No learning curve |
| **Tibial angle** |  |  |  |  |  |  |  |  |
| BMI < 27 | -2.1 ±2.71 | -0.7 ±1.89 | -0.75 ±0.97 | - | -0.55 ±0.89 | Ref | 0.29 (p=0.004*) | The learning curve stabilized after the 18th patient group |
| BMI ≥ 27 | -3.1 ±2.07 | -1.75 ±2.24 | -0.85 ±1.46 | -0.35 ±0.75 | -0.6 ±0.94 | -0.26 (p=0.396) | 0.32 (p<0.001*) | The learning curve stabilized after the 23th patient group |
| **Femoral angle** |  |  |  |  |  |  |  |  |
| BMI < 27 | 0.3 ±3.63 | -0.05 ±2.39 | 0.35 ±1.79 | - | 0.7 ±1.45 | Ref | -0.01 (p=0.903) | No learning curve |
| BMI ≥ 27 | 0.25 ±3.43 | 0.35 ±2.80 | 0.2 ±2.33 | 0.35 ±1.27 | -0.3 ±1.66 | -0.46 (p=0.310) | -0.09 (p=0.327) | No learning curve |
| **Tibial slope** |  |  |  |  |  |  |  |  |
| BMI < 27 | 2.35 ±2.58 | 3.8 ±2.19 | 5.5 ±1.05 | - | 5.5 ±1.19 | Ref | 0.47 (p<0.001*) | The learning curve stabilized after the 26th patient group |
| BMI ≥ 27 | 4.2 ±2.48 | 3.4 ±2.62 | 5.1 ±0.91 | 5.45 ±1.0 | 5.6 ±0.82 | 0.57 (p=0.087) | 0.27 (p=0.003*) | The learning curve stabilized after the 25th patient group |
| **Anterior protrusion** |  |  |  |  |  |  |  |  |
| BMI < 27 | 0.2 ±0.41 | 0.1 ±0.31 | -0.05 ±0.51 | - | -0.1 ±0.45 | Ref | -0.25 (p=0.023*) | Linear decrease of 0.003 (SE=0.001) for each patient group |
| BMI ≥ 27 | 0.3 ±0.57 | -0.15 ±0.59 | -0.05 ±0.22 | 0 ± 0 | 0 ±0 | -0.06 (p=0.388) | -0.18 (p=0.067) | No learning curve |
| **Posterior protrusion** |  |  |  |  |  |  |  |  |
| BMI < 27 | -0.75 ±1.12 | -0.25 ±0.64 | 0.05 ±0.22 | - | 0.05 ±0.22 | Ref | 0.38 (p=<0.001*) | The learning curve stabilized after the 21th patient group |
| BMI ≥ 27 | -0.3 ±0.92 | 0.2 ±0.69 | 0.05 ±0.69 | 0.05 ±0.51 | 0 ±0.56 | 0.22 (p=0.080) | 0.14 (p=0.145) | No learning curve |
| **Medial protrusion** |  |  |  |  |  |  |  |  |
| BMI < 27 | -0.15 ±0.90 | 0.05 ±0.83 | -0.1 ±0.72 | - | -0.15 ±0.75 | Ref | 0.03 (p=0.756) | No learning curve |
| BMI ≥ 27 | 0.05 ±0.83 | 0.15 ±0.67 | 0.05 ±0.39 | 0.05 ±0.39 | -0.2 ±0.70 | 0.15 (p=0.267) | -0.14 (p=0.143) | No learning curve |
| **Bearing (thickness)** |  |  |  |  |  |  |  |  |
| BMI < 27 | 8.5 ±0.83 | 8.25 ±0.55 | 8.35 ±0.75 | - | 8.35 ±0.75 | Ref | -0.08 (p=0.443) | No learning curve |
| BMI ≥ 27 | 8.4 ±0.50 | 8.3 ±0.47 | 8.4 ±0.50 | 8.45 ±0.95 | 8.45 ±0.95 | -0.02 (p=0.898) | -0.10 (p=0.326) | No learning curve |

*=statistical significant value (p<0.05); Ref= reference group; BMI=Body Mass Index.

**Table S4.** Learning curve by side: right (n=61, 42 patient groups) and left side (n=64, 45 patient groups).

|  | ***Mean ±SD*** | | | | **Between group test**  ***Mean difference (p-value)*** | **Subgroup analysis** | |
| --- | --- | --- | --- | --- | --- | --- | --- |
|  | **1^st^ patient group** | **15^th^ patient group** | **30^th^ patient group** | **last patient group** |  | **Mann–Kendall trend test *Value (p-value)*** | **Learning curve** |
| **Surgical time** |  |  |  |  |  |  |  |
| Right side | 49.4 ±8.57 | 51 ±7.18 | 45.5 ±8.22 | 42.2 ±10.0 | Ref | -0.21 (p=0.016*) | Linear decrease of 0.11 (SE=0.04) for each patient group after the 14^th^ patient group |
| Left side | 49.2 ±10.7 | 49.8 ±9.83 | 50.6 ±9.61 | 44.6 ±10.4 | 1.21 (p=0.474) | -0.10 (p=0.273) | No learning curve |
| **Tibial angle** |  |  |  |  |  |  |  |
| Right side | -2.25 ±2.38 | -0.8 ±1.40 | -1 ±1.30 | -0.55 ±0.95 | Ref | 0.3 (p=0.002*) | The learning curve stabilized after the 13th patient group |
| Left side | -2.75 ±2.15 | -1.8 ±2.75 | -0.45 ±0.99 | -0.6 ±0.88 | 0.16 (p=0.611) | 0.30 (p=0.001*) | The learning curve stabilized after the 27th patient group |
| **Femoral angle** |  |  |  |  |  |  |  |
| Right side | 0.05 ±2.33 | 0.25 ±2.57 | -0.05 ±1.93 | -0.15 ±1.63 | Ref | -0.11 (p=0.258) | No learning curve |
| Left side | 0.85 ±4.03 | -0.35 ±3.27 | 0.15 ±1.69 | 0.55 ±1.57 | 0.01 (p=0.980) | -0.01 (p=0.916) | No learning curve |
| **Tibial slope** |  |  |  |  |  |  |  |
| Right side | 3.05 ±2.54 | 4.45 ±1.96 | 5.6 ±1.05 | 5.55 ±0.99 | Ref | 0.39 (p<0.001*) | The learning curve stabilized after the 23th patient group |
| Left side | 3.8 ±2.84 | 3.15 ±2.43 | 4.9 ±1.02 | 5.55 ±1.05 | -0.19 (p=0.563) | 0.32 (p<0.001*) | The learning curve stabilized after the 33th patient group |
| **Anterior protrusion** |  |  |  |  |  |  |  |
| Right side | 0.2 ±0.70 | -0.15 ±0.49 | 0 ±0 | 0 ±0 | Ref | -0.28 (p=0.007*) | The learning curve stabilized after the 25th patient group |
| Left side | 0.2 ±0.52 | 0.05 ±0.39 | 0 ±0.56 | -0.1 ±0.45 | -0.05 (p=0.524) | -0.17 (p=0.105) | No learning curve |
| **Posterior protrusion** |  |  |  |  |  |  |  |
| Right side | -0.55 ±0.67 | -0.1 ±0.72 | -0.05 ±0.51 | 0 ±0.32 | Ref | 0.29 (p=0.005*) | The learning curve stabilized after the 15th patient group |
| Left side | -0.45 ±1.28 | 0 ±0.73 | 0.05 ±0.22 | 0.05 ±0.51 | 0.12 (p=0.321) | 0.21 (p=0.037*) | Linear increase of 0.007 (SE=0.003) for each patient group |
| **Medial protrusion** |  |  |  |  |  |  |  |
| Right side | -0.15 ±0.81 | 0 ±0.80 | -0.05 ±0.61 | -0.25 ±0.72 | Ref | -0.05 (p=0.647) | No learning curve |
| Left side | 0.15 ±0.93 | 0.3 ±0.98 | 0 ±0.56 | -0.1 ±0.72 | 0.20 (p=0.136) | -0.04 (p=0.659) | No learning curve |
| **Bearing (thickness)** |  |  |  |  |  |  |  |
| Right side | 8.25 ±0.44 | 8.45 ±0.61 | 8.35 ±0.67 | 8.5 ±1.05 | Ref | -0.02 (p=0.839) | No learning curve |
| Left side | 8.5 ±0.76 | 8.4 ±0.60 | 8.15 ±0.37 | 8.3 ±0.57 | -0.04 (p=0.731) | -0.15 (p=0.137) | No learning curve |

*=statistical significant value (p<0.05); Ref= reference group.

**Table S5.** Learning curve by tibial size: small (size C-D-E, n=73, 54 patient groups) and big size (size F-G-H-J, n=52, 33 patient groups).

|  | ***Mean ±SD*** | | | | | **Between group test**  ***Mean difference (p-value)*** | **Subgroup analysis** | |
| --- | --- | --- | --- | --- | --- | --- | --- | --- |
|  | **1^st^ patient group** | **15^th^ patient group** | **30^th^ patient group** | **45^th^ patient group** | **last patient group** |  | **Mann–Kendall trend test *Value (p-value)*** | **Learning curve** |
| **Surgical time** |  |  |  |  |  |  |  |  |
| Small size | 48.4 ±9.42 | 51.2 ±7.60 | 49.8 ±7.50 | 43.8 ±8.63 | 41.8 ±9.22 | Ref | -0.14 (p=0.087) | No learning curve |
| Big size | 50.7 ±10.4 | 50.6 ±9.81 | 48.4 ±10.2 | - | 44.9 ±11.1 | 1.51 (p=0.380) | -0.17 (p=0.078) | No learning curve |
| **Tibial angle** |  |  |  |  |  |  |  |  |
| Small size | -2.95 ±1.73 | -1.95 ±2.44 | -0.7 ±1.53 | -0.65 ±0.93 | -0.65 ±0.93 | Ref | 0.35 (p<0.001*) | The learning curve stabilized after the 27th patient group |
| Big size | -2.15 ±2.62 | -0.6 ±1.43 | -0.3 ±0.73 | - | -0.5 ±0.89 | 0.12 (p=0.696) | 0.20 (p=0.060) | No learning curve |
| **Femoral angle** |  |  |  |  |  |  |  |  |
| Small size | 1.05 ±3.44 | 0.9 ±3.23 | 0.85 ±2.43 | 0.15 ±1.50 | 0.2 ±1.58 | Ref | -0.06 (p=0.508) | No learning curve |
| Big size | -0.1 ±3.02 | -0.3 ±1.56 | 0.5 ±1.32 | - | 0.2 ±1.70 | -0.18 (p=0.690) | 0.01 (p=0.941) | No learning curve |
| **Tibial slope** |  |  |  |  |  |  |  |  |
| Small size | 4.2 ±1.94 | 3.5 ±2.37 | 4.4 ±1.14 | 5.6 ±1.14 | 5.5 ±1.0 | Ref | 0.33 (p<0.001*) | The learning curve stabilized after the 33th patient group |
| Big size | 2.9 ±3.19 | 5.15 ±0.81 | 5.45 ±1.19 | - | 5.6 ±1.05 | -0.22 (p=0.527) | 0.40 (p<0.001*) | The learning curve stabilized after the 17th patient group |
| **Anterior protrusion** |  |  |  |  |  |  |  |  |
| Small size | 0.35 ±0.59 | 0.1 ±0.32 | 0 ±0.32 | 0.05 ±0.22 | 0.1 ±0.45 | Ref | -0.023 (p=0.017*) | The learning curve stabilized after the 34th patient group |
| Big size | 0.1 ±0.45 | 0.05 ±0.22 | -0.1 ±0.45 | - | -0.1 ±0.45 | -0.03 (p=0.678) | -0.17 (p=0.132) | No learning curve |
| **Posterior protrusion** |  |  |  |  |  |  |  |  |
| Small size | -0.75 ±1.07 | -0.15 ±0.75 | 0.1 ±0.45 | 0.05 ±0.22 | 0 ±0.32 | Ref | 0.38 (p<0.001*) | The learning curve stabilized after the 23th patient group |
| Big size | -0.15 ±0.93 | -0.2 ±0.70 | 0.05 ±0.51 | - | 0.05 ±0.51 | 0.08 (p=0.515) | 0.05 (p=0.668) | No learning curve |
| **Medial protrusion** |  |  |  |  |  |  |  |  |
| Small size | 0.05 ±0.95 | 0.25 ±1.07 | 0.1 ±0.64 | 0.05 ±0.39 | 0.05 ±0.39 | Ref | -0.01 (p=0.931) | No learning curve |
| Big size | -0.05 ±0.56 | 0 ±0.56 | -0.2 ±0.70 | - | -0.4 ±0.88 | -0.23 (p=0.093) | -0.14 (p=0.225) | No learning curve |
| **Bearing (thickness)** |  |  |  |  |  |  |  |  |
| Small size | 8.55 ±0.76 | 8.3 ±0.57 | 8.35 ±0.49 | 8.2 ±0.52 | 8.3 ±0.55 | Ref | -0.16 (p=0.093) | No learning curve |
| Big size | 8.3 ±0.47 | 8.25 ±0.55 | 8.55 ±1.05 | - | 8.55 ±1.05 | 0.08 (p=0.532) | 0.03 (p=0.803) | No learning curve |

*=statistical significant value (p<0.05).

**Table S6.** Learning curve by femoral size: small (size 1-2-3-4, n=77, 58 patient groups) and big size (size 5-6-7, n=48, 29 patient groups).

|  | ***Mean ±SD*** | | | | | **Between group test**  ***Mean difference (p-value)*** | **Subgroup analysis** | |
| --- | --- | --- | --- | --- | --- | --- | --- | --- |
|  | **1^st^ patient group** | **15^th^ patient group** | **30^th^ patient group** | **45^th^ patient group** | **last patient group** |  | **Mann–Kendall trend test *Value (p-value)*** | **Learning curve** |
| **Surgical time** |  |  |  |  |  |  |  |  |
| Small size | 45.7 ±7.33 | 47.9 ±8.16 | 49.7 ±9.48 | 45.3 ±8.27 | 43.0 ±9.19 | Ref | -0.09 (p=0.258) | No learning curve |
| Big size | 51.8 ±10.9 | 50.8 ±9.03 | - | - | 43.8 ±11.3 | 2.31 (p=0.190) | -0.25 (p=0.014*) | Linear decrease of 0.10 (SE=0.04) for each patient group |
| **Tibial angle** |  |  |  |  |  |  |  |  |
| Small size | -3.1 ±2.17 | -1.95 ±2.37 | -0.7 ±1.17 | -0.75 ±0.79 | -0.6 ±0.75 | Ref | 0.30 (p<0.001*) | The learning curve stabilized after the 22th patient group |
| Big size | -2.65 ±2.62 | -1.2 ±2.46 | - | - | -0.55 ±1.05 | -0.05 (p=0.890) | 0.29 (p=0.008*) | The learning curve stabilized after the 20th patient group |
| **Femoral angle** |  |  |  |  |  |  |  |  |
| Small size | 0.45 ±3.73 | 0.35 ±3.15 | 0.5 ±2.31 | 0.4 ±1.70 | 0.5 ±1.61 | Ref | -0.02 (p=0.844) | No learning curve |
| Big size | -0.05 ±3.30 | -0.45 ±2.21 | - | - | -0.1 ±1.62 | -0.29 (p=0.539) | 0.19 (p=0.365) | No learning curve |
| **Tibial slope** |  |  |  |  |  |  |  |  |
| Small size | 3.7 ±2.45 | 3.15 ±2.54 | 4.7 ±1.26 | 5.6 ±1.05 | 5.65 ±0.88 | Ref | 0.38 (p<0.001*) | The learning curve stabilized after the 35th patient group |
| Big size | 3 ±2.96 | 4.15 ±2.23 | - | - | 5.45 ±1.15 | -0.25 (p=0.481) | 0.34 (p=0.002*) | The learning curve stabilized after the 20th patient group |
| **Anterior protrusion** |  |  |  |  |  |  |  |  |
| Small size | 0.25 ±0.55 | 0.05 ±0.39 | 0.05 ±0.39 | 0.05 ±0.22 | 0 ±0 | Ref | -0.17 (p=0.066) | No learning curve |
| Big size | 0.1 ±0.72 | -0.25 ±0.64 | - | - | -0.1 ±0.45 | -0.08 (p=0.270) | -0.27 (p=0.116) | No learning curve |
| **Posterior protrusion** |  |  |  |  |  |  |  |  |
| Small size | -0.35 ±0.99 | 0 ±0.86 | 0.1 ±0.45 | 0.2 ±0.52 | 0.1 ±0.55 | Ref | 0.23 (p=0.012*) | The learning curve stabilized after the 17th patient group |
| Big size | -0.6 ±1.1 | -0.25 ±0.72 | - | - | -0.05 ±0.22 | -0.24 (p=0.067) | 0.27 (p=0.020*) | Linear increase of 0.01 (SE=0.003) for each patient group |
| **Medial protrusion** |  |  |  |  |  |  |  |  |
| Small size | -0.05 ±0.99 | 0.2 ±1.06 | 0 ±0.32 | 0.05 ±0.39 | 0 ±0.32 | Ref | -0.06 (p=0.532) | No learning curve |
| Big size | -0.1 ±0.85 | -0.1 ±0.79 | - | - | -0.35 ±0.93 | -0.27 (p=0.057) | -0.05 (p=0.654) | No learning curve |
| **Bearing (thickness)** |  |  |  |  |  |  |  |  |
| Small size | 8.7 ±0.80 | 8.35 ±0.59 | 8.3 ±0.47 | 8.1 ±0.31 | 8.35 ±0.59 | Ref | -0.17 (p=0.074) | No learning curve |
| Big size | 8.2 ±0.41 | 8.35 ±0.59 | - | - | 8.45 ±1.05 | 0.00 (p=0.999) | 0.032 (p=0.792) | No learning curve |

*=statistical significant value (p<0.05); Ref= reference group.
